# Molecular identification of potentially pathogenic free-living amoebae in environmental samples from urban areas of Kerman, Southeastern Iran

**DOI:** 10.1016/j.parepi.2026.e00476

**Published:** 2026-01-03

**Authors:** Abbas Ali Ghasemi, Elham Akhlaghi, Zahra Babaei, Naser Zia-ali, Sima Rostami, Mehdi Borhani, Tayyebeh Ghasemi, Majid Fasihi Harandi

**Affiliations:** a Research Center for Hydatid Disease in Iran, Institute of Infectious Diseases and Tropical Medicine, Department of Parasitology, Afzalipour School of Medicine, Kerman University of Medical Sciences, Kerman, Iran; b Leishmaniasis Research Center, Institute of Infectious Diseases and Tropical Medicine, Afzalipour School of Medicine, Kerman University of Medical Sciences, Kerman, Iran; c Research Center for Tropical and Infectious Diseases, Institute of Infectious Diseases and Tropical Medicine, Kerman University of Medical Sciences, Kerman, Iran; d LifeLabs Medical Laboratories, Toronto, Ontario, Canada; e State Key Laboratory of Pathogenesis, Prevention and Treatment of High Incidence Diseases in Central Asia, Xinjiang Medical University, Urumqi, China; f Department of Animal Biology, Faculty of Natural Sciences, University of Tabriz, Tabriz, Iran

**Keywords:** *Acanthamoeba*, *Balamuthia*, Free living amoebae (FLA), Soil, *Vermamoeba*, Water

## Abstract

Free-living amoebae (FLA) are widely distributed protozoans in various habitats. Some genera of these amoebae have the potential to be pathogenic to humans. The aim of this study was to identify the distribution of different FLA species in various environmental sources in Kerman, southeastern Iran. A total of 141 samples, including city fountains, tap water, and soil samples, were collected from various regions of the city. After filtration and cultivation on non-nutrient agar, molecular analysis was performed using PCR-sequencing with genus specific primers for *Acanthamoeba*, *Balamuthia, Vahlkampfidae*, and *Hartmannella*. PCR- sequencing fountain water samples revealed the presence of *Acanthamoeba*, *Hartmannella* and *Naegleria* species. Among the sequences obtained from *Acanthamoeba* isolates, 72.7 %, 18.2 %, and 9.1 % belonged to the genotype T4, T3, and T6, respectively. Two *Naegleria* species were identified as *Naegleria canariensis* and *N. australiensis*. Molecular analysis of tap water samples showed the presence of *Acanthamoeba* T6 genotype and *Hartmannella (Vermamoeba) vermiformis*. Sequencing of soil samples revealed the presence of *Acanthamoeba lenticulata* (T5) and *Vermamoeba vermiformis* species. The presence of potentially pathogenic FLA in urban environments in Iran presents potential hazards of infection for the people and appropriate measures needed to minimize the risks to human health including disinfection of water systems using chlorine dioxide, molecular monitoring, the implementation of educational measures for the public and health professionals.

## 1 Introduction

Free-living amoebae (FLA) are a group of protozoa universally found in diverse environments, including seawater, freshwater lakes, pool water, hot springs, saltwater lakes, soil, dust, and air (Gianinazzi et al., 2010; Badirzadeh et al., 2011; Chaúque et al., 2023; Chaúque et al., 2022). Several genera of FLA have been identified with pathogenic potential to human and animals including *Acanthamoeba*, *Balamuthia*, *Naegleria, Sappinia*, *Vermamoeba,* and *Paravahlkampfia*. Numerous reports of the pathogenicity of FLA are published, however due to the poor diagnosis and lack of awareness in medical professionals, the true incidence of human infections is probably under-estimated (Bellini et al., 2018; Kofman and Guarner, 2022).

Among these amoebae, *Acanthamoeba*, and *Naegleria fowleri* are the most common causes of infections (Visvesvara et al., 2009) that can potentially lead to serious infections through involvement of the central nervous system (CNS) and other organs. A wide range of environmental matrices, including air, freshwater bodies, saline habitats, dust, sediments, and soil, as well as various anthropogenic settings such as ventilation systems, public swimming pools, bottled water, contact lenses, dialysis units, and dental instruments, have been implicated as potential sources of transmission for FLA. The transmission is mainly through direct inoculation into the skin, penetration into the ocular tissues, or inhalation into the respiratory tract, and consequently lead to disease at these sites of entry (Hendiger-Rizo et al., 2024; Kofman and Guarner, 2022). FLA also serve as reservoirs of various bacterial endosymbionts many of them pathogenic to human and contributing to the development of virulence traits and enhancing microbial resistance. These properties pose significant challenges to therapeutic interventions and underscore the ecological role of FLA in pathogen dissemination (Balczun and Scheid, 2017). Central nervous system (CNS) infections caused by FLA, such as primary amoebic meningoencephalitis (PAM) and granulomatous amoebic encephalitis (GAE), remain extremely difficult to manage and are associated with mortality rates exceeding 90 %, highlighting the substantial therapeutic challenges encountered in managing diseases caused by FLA. Diagnostic delays are common because clinical manifestations are non-specific and often mimic viral or bacterial meningitis, allowing rapid disease progression and frequently leading to death within a short period. Treatment options are further constrained by the limited ability of most therapeutic agents to cross the blood–brain barrier. This challenge is exemplified by the suboptimal CNS bioavailability of Amphotericin B, the principal therapeutic agent used for amebic encephalitis. (Güémez and García, 2021; Spottiswoode et al., 2024).

Most FLA species form cysts to remain dormant under harsh environmental conditions such as long starvation, desiccation, UV radiation and fluctuations changes in temperature and pH levels (Fouque et al., 2012). *Acanthamoeba* species are the most ubiquitous FLA with infections commonly reported from various regions around the world (Niyyati et al., 2014). *Acanthamoeba* species are classified into 23 genotypes (T1–T23) based on their 18S rRNA sequences (Putaporntip et al., 2021). Various studies have shown certain genotypes of *Acanthamoeba,* particularly the T4 genotype are responsible for most human pathologies in human including granulomatous amoebic encephalitis (GAE), skin ulcers and upper respiratory tract infection in immunocompromised patients (Khan, 2006) as well as amoebic keratitis (AK) in healthy individuals (Lorenzo-Morales et al., 2015). Similar cases of AK have also been reported involving *Vermamoeba* and other Vahlkampfiid amoeba (Abedkhojasteh et al., 2013). Several studies on clinical and environmental samples from Iran have identified T2, T3, T4 and T11 genotypes of *Acanthamoeba* (Maghsood et al., 2005; Niyyati et al., 2009b). Among the more than 47 *Naegleria* species, only *N. fowleri* is known to cause Primary Amoebic Meningoencephalitis (PAM) in humans (Latifi et al., 2017). Results of a study on therapeutic geothermal water sources in north of Iran showed 54 % of the examined water samples were positive for six *Naegleria* species. The most identified species was *N. australiensis*, however the pathogenic *N. fowleri* was not detected (Latifi et al., 2017). One study in Markazi province of Iran revealed the presence of *Acanthamoeba* (T4 and T5 genotypes), *Naegleria (N. australiensis*, *N. pagei*, and *N. gruberi*), *V. vermiformis*, *Vannella* sp., *Vahlkampfia avara*, and *Stenamoeba polymorpha* in water sources (Fani et al., 2022).

*Balamuthia mandrillaris* is known to cause *Balamuthia* Amoebic Encephalitis (BAE), GAE, and disseminated skin infections (Siddiqui and Khan, 2015). Some studies in north, northwestern and central of Iran showed the presence of *B. mandrillaris* from different environmental samples including water, soil, and dust (Latifi et al., 2016; Niyyati et al., 2009b; Niyyati et al., 2009a).

Our knowledge on the frequency of different FLA species in southeastern of Iran is limited to several studies. Three genotypes of *Acanthamoeba* (T3, T4 and T5) were found in pools and surface water samples in Zabol (Aghajani et al., 2016). A recent study investigated different *Acanthamoeba* species in public swimming pools from south of Kerman province (Eftekhari-Kenzerki et al., 2021). The purpose of the present study was to investigate the occurrence of different potentially pathogenic FLA species in various environmental sources including soil, tap and fountain waters in Kerman city, southeastern of Iran using morphological and molecular tools. We have also reviewed the current situation and public health implications of FLA in southeast of the country.

## 2 Materials and methods

### 2.1 Study location

Kerman, the largest city in southeast of Iran, is the capital city of Kerman province, the largest province of Iran with a population of approximately 3.164 million people. The city is situated at an elevation of 1749 m (5,738 ft) above sea level with a population of 537,718 as of the 2016 census. Environmental samples were collected from various places in the city of Kerman. The city was divided into 5 zones, including north, south, east, west and central areas. The samples were collected from tap water, fountain water and soil in public parks, squares, and public gardens. The fountain water and soil samples were collected from a depth of 10 cm and 5 cm, respectively. Finally, all the samples were immediately transported to the research laboratory for further morphological and molecular studies.

### 2.2 Samples and sampling

**Fig. 1:**
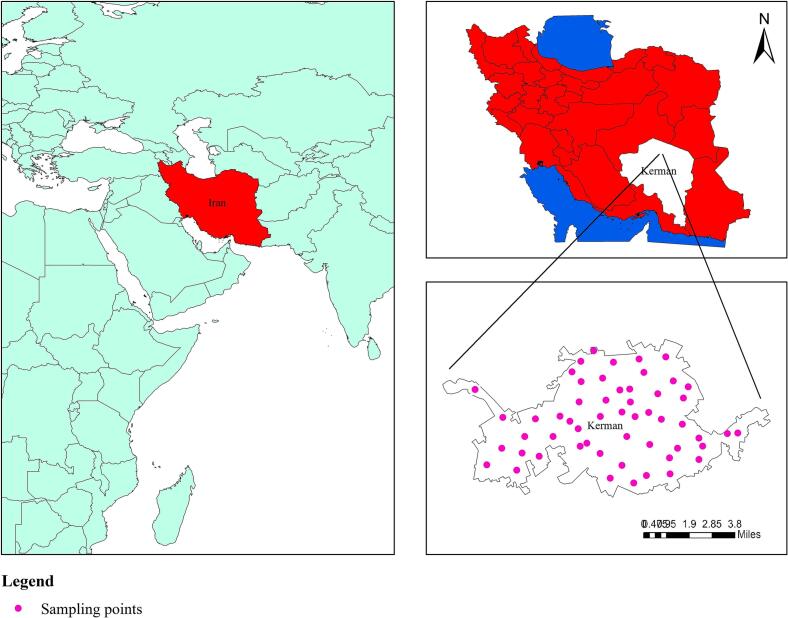
A schematic map illustrating the study area where samples were collected from various urban locations in Kerman, southeastern Iran.

A total of 141 environmental samples were systematically collected from various urban locations across Kerman, the capital city of Kerman Province in southeastern Iran (Fig. 1). These included 91 fountain water samples, 34 soil samples and 16 tap water samples. To achieve comprehensive spatial coverage, the city was divided into five geographic zones, i.e. North, South, East, West, and Central. Environmental samples were then collected from a variety of public settings within these zones, including parks, squares, and gardens. Sampling was conducted with standardized protocols applied across all matrices. Fountain water was collected directly from surface outlets using sterile 500 mL bottles. Soil samples were obtained from a uniform depth of 5 cm using trowels, with 100–200 g of material collected per sample. Tap water samples were similarly collected in 500 mL volumes from public parks. All specimens were promptly transported to the laboratory under controlled conditions. Water samples were maintained at 4–8 °C, while soil samples were sealed in bags to prevent desiccation.

### 2.3 Filtration, culture, and morphological analysis

Approximately 500 mL of each water sample was filtrated through an autoclaved 0.45 μm pore size cellulose nitrate membrane (Whatman, Grade 42, UK) under a vacuum (Solgi et al., 2012). About 100–200 g of each soil sample were suspended in sterile distilled water, remained for about an hour, and then filtered as mentioned above. The membrane filters were aseptically placed on to Petri dishes containing 1.5 % non-nutrient agar (NNA) overlaid with a uniform layer of heat-killed *Escherichia coli*. The bacterial suspension was prepared by autoclaving at 121 °C for 15 min to ensure complete inactivation. Using sterile forceps, the filters were carefully positioned face-down and lightly pressed onto the agar surface to ensure optimal contact and facilitate amoebic migration. For each filtered sample, three plates were incubated at 28 °C, 30 °C, and 40 °C for up to 30 days. These temperatures reflect typical environmental conditions for isolating and maintaining FLA (28 °C), support trophozoite proliferation and indicate thermotolerance relevant to pathogenicity (30 °C), and assess high-temperature tolerance, a key criterion for identifying potentially pathogenic strains (40 °C) (Rayamajhee et al., 2022; Wang et al., 2023).

The plates were examined daily under an optical microscope to monitor the presence, motility, and morphological characteristics of FLA. Subsequently, to further ensure the purity of amoebic isolates and promoting clonal expansion and to minimize fungal and bacterial contamination, all cultures that tested positive were subsequently sub-cultured onto fresh NNA plates supplemented with heat-killed *E. coli* using a sterile swab and incubated for an additional 14 days. Amoebae were carefully collected from clean areas of positive plates using a sterile loop (Akhlaghi et al., 2025; Latifi et al., 2020; Montalbano Di Filippo et al., 2015).

### 2.4 PCR-sequencing and genotype identification

To collect FLA, approximately 500 μL of phosphate-buffered saline (PBS; pH 7.2) was added to each culture plate. The surface of the culture medium was gently washed and scraped using a sterile loop to dislodge the amoebae. The resulting suspension was carefully transferred into sterile microtubes containing PBS. To concentrate the organisms, the suspension was centrifuged at 8000 rpm for 2 min. DNA was subsequently extracted from the pellet and stored at −20 °C until PCR analysis.

The DNA extraction was performed using High Pure PCR Template Preparation kit (Roche, Germany) according to the manufacturer's instructions. DNA concentrations were measured using a NanoDrop spectrophotometer (NanoDrop™ 1000, Thermo Fisher Scientific, Waltham, MA, USA) and stored at −20 °C until use.

As shown in Table 1 four sets of primers were used to amplify specific target genes. All the primer sets were commercially provided by Macrogen, Seoul, Korea. PCR reactions were performed in a thermal cycler (Analytik Jena, Jena, Germany) in a final volume of 25 μL containing 8 μL Taq Master Mix (Cinnagen, Iran), 1 μL template DNA, 1 μL of each primer and 14 μL distilled water (Supplementary Table 1). Finally, PCR products were electrophoresed by using 2 % agarose gel and visualized under UV gel Doc system (UVitec).

PCR products were sequenced by a commercial sequencing company (Macrogen, Seoul, Korea). Sequencing data were compared with the published sequences in GenBank using NCBI BLAST system. Sequence alignments were performed by ClustalW multiple sequence alignment tool from BioEdit v7.0.9. Phylogenetic analyses were performed using neighbor joining method with 1000 bootstrap values by MEGA 7.0 software.

**Table 1:** The primer sets used for molecular identification of free-living amoeba from various environmental sources in the city of Kerman, southeastern Iran.

FLA	Primer sequence (5′ to 3′)	Target gene	Amplicon size (bp)
*Acanthamoeba* spp.	JDP1 (F): GGCCCAGATCGTTTACCGTGAA JDP2 (R): CTCACAAGCTGCTAGGGAGTCA	18S rRNA	500
*Hartmannella (Vermamoeba)*	NA1 (F): TTACGAGGTCAGGACACTGT NA2 (R): GACCATCCGGAGTTCTCG	18S rRNA	500
*Vahlkampfidae*	ITS1 (F): GTCTTCGTAGGTGAACCTGC ITS2 (R): CCGCTTACTGATATGCTTAA	5.8S rRNA	500
*Balamuthia mandrillaris*	F: CGCATGTATGAAGAAGACCA R: TTACCTATATAATTGTCGATACCA	18S rRNA	1075

## 3 Results

Overall, 141 samples were collected from various places in the city of Kerman. The samples were collected from tap water (16), fountain water (91) and soil (34) in public parks, squares, and public gardens. FLA were observed in a total of 73 out of 141 (51.8 %) specimens from different environmental sources (fountain water, soil and tap water). Among the 73 culture-positive samples, 48 isolates yielded PCR product, of which 43 samples were randomly selected for sequencing. Ultimately, 34 isolates were successfully sequenced including 20 from fountain water, 12 from soil, and 2 from tap water (Table 2). The sequences were submitted to the NCBI GenBank with the accession numbers KC694162- KC694192 and OP218467- OP218468.

**Table 2:** Frequency distribution of free-living amoebae from different environmental samples using parasitological and molecular methods.

Sample source	Culture positive, no. (%)	PCR positive / PCR examined, no. (%)	Successful sequencing / no. of sequences (%)	*Acanthamoeba* (Sequencing)*,* no. (%)	*Hartmannella* (Sequencing)*,* no. (%)	*Naegleria (*Sequencing)*,* no. (%)
Fountain water	49/91 (53.8)	28/36 (77.7)	20/24 (83.3)	11/20 (55)	7/20 (35)	2/20 (10)
Tap water	3/16 (18.8)	3/3 (100)	2/3 (66.6)	1/2 (50)	1/2 (50)	Negative
Soil	21/34 (61.8)	17/21 (80.1)	12/16 (75)	4/12 (33.33)	8/12 (66.6)	Negative
Total	73/141 (51.7)	48/60 (80)	34/43 (79.1)	16/34 (47.1)	16/34 (47.1)	2/34 (5.88)

### 3.1 Culture and molecular identification of FLA from water samples

**Fig. 2:**
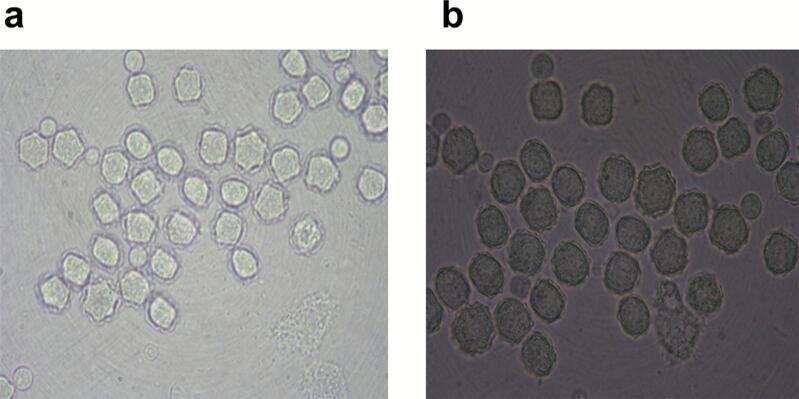
Microscopic view of *Acanthamoeba* cultured on non-nutrient agar (NNA). (A) cysts, (B) trophozoites**.**

**Supplementary Fig. 1:**
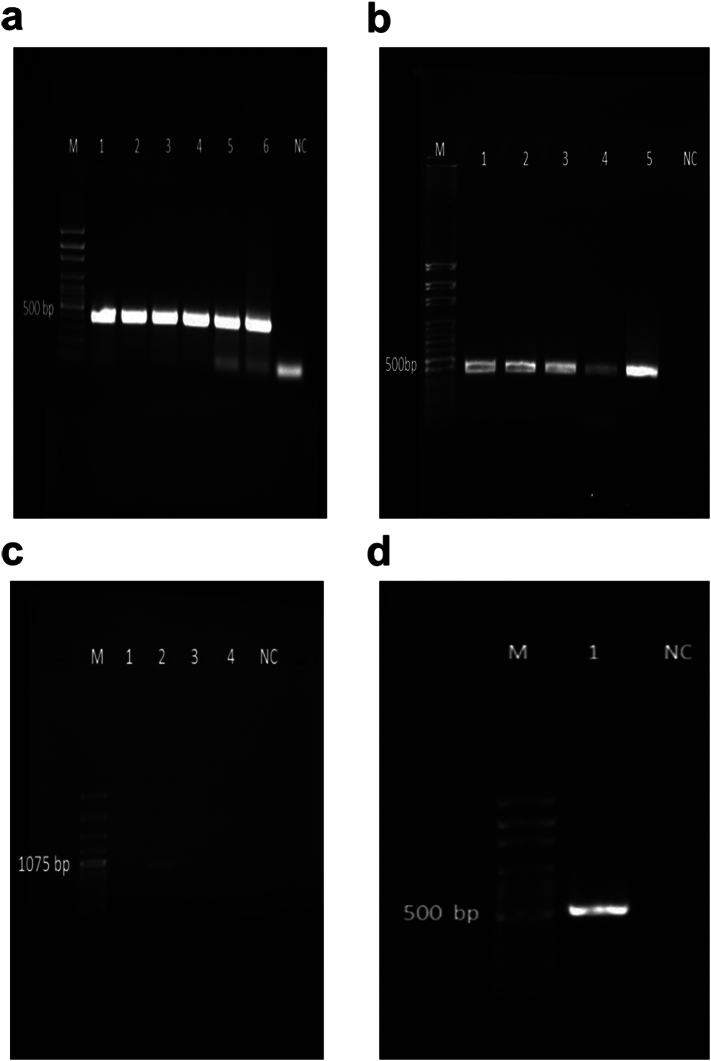
Agarose gel electrophoresis of PCR products of ribosomal RNA region. a) *Acanthamoeba*, b) *Hartmannella* (*Vermamoeba*), c) *Naegleria*, d) *Balamuthia*. M: DNA size marker, NC: Negative Control.

Using the culture method, 49 out of 91 fountain water samples (53.8 %) tested positive for FLA (Fig. 2). These culture-positive samples were further analyzed using molecular methods. Out of the 49 culture-positive samples, 36 were chosen for DNA extraction. As summarized in Table 2, PCR on 28 out of 36 samples (77.7 %) tested positive for FLA, with *Acanthamoeba* spp. found in 60.7 % (17/28), *Hartmannella* in 28.6 % (8/28), and *Vahlkampfidae* in 10.7 % (3/28) of the samples (Supplementary Fig. 1). None of the samples showed amplification using *Balamuthia* specific primer sets. To characterize the ameba, sequencing was conducted on 24 PCR-positive samples, including 14 *Acanthamoeba*, 8 *Hartmannella*, and 2 *Vahlkampfidae* PCR amplicons.

Among *Acanthamoeba* sequences, 72.7 % (8/11), 18.2 % (2/11), and 9.1 % (1/11) of the isolates belonged to genotype T4 (*A. castellanii, A. polyphaga*), genotype T3 (*A. griffini*), and genotype T6 (*A. palestinensis*), respectively. Additionally, sequencing analysis of 8 *Hartmannella* samples identified 7 samples as *H. (Vermamoeba) vermiformis.* The two *Vahlkampfidae* samples were identified as *Naegleria canariensis* and *N. australiensis*.

FLA growth was observed in 18.8 % (3/16) of tap water samples (Fig. 2). PCR results of culture-positive samples showed the presence of two *Acanthamoeba* and one *Hartmannella* species (Supplementary Fig. 1). The sequencing of the two PCR-positive *Acanthamoeba* identified one isolate as genotype T6 (*A. palestinensis*). Additionally, the PCR-sequencing of *Hartmannella* showed this sample belonged to the *H. (Vermamoeba) vermiformis* (Table 2).

### 3.2 Culture and molecular detection of FLA from soil samples

In 61.8 % (21/34) of soil samples, FLA growth was observed (Fig. 2). PCR results indicated a positive of 80.1 % (17/21) for FLA, including *Acanthamoeba* (7/17, 41.2 %), *Hartmannella* (9/17, 52.9 %), and *Balamuthia* (1/17, 5.88 %) species. Sequencing was performed on 16 selected PCR positive samples, comprising 6 *Acanthamoeba* spp., 9 *Hartmannella*, and one *Balamuthia.* Molecular analysis of the *Acanthamoeba* isolates revealed that 4 out of 6 samples belonged to the T5 genotype (*A. lenticulata*). Eight out of 9 *Hartmannella* isolates (88.9 %) were identified as *H. (Vermamoeba) vermiformis*. Sequencing analysis of the *Balamuthia* did not yield good results (Table 2).

**Fig. 3:**
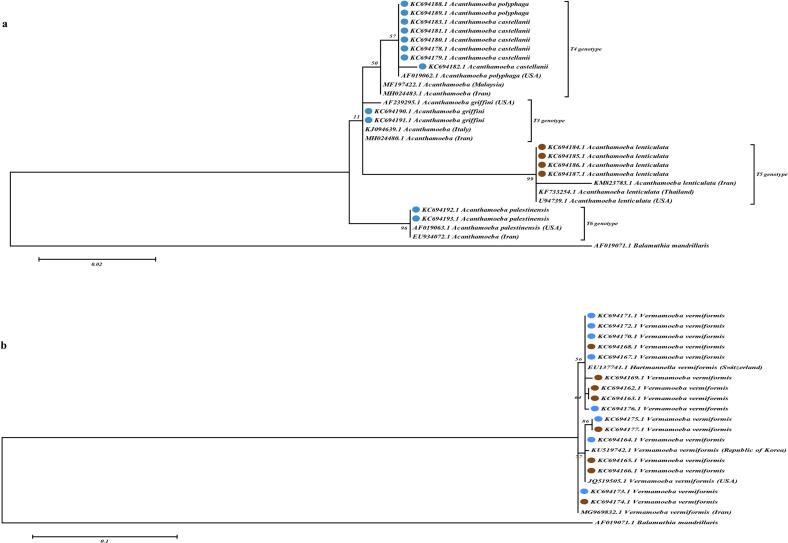
Phylogenetic relationships of *Acanthamoeba* spp. (A) and *Hartmannella* (*Vermamoeba*) *vermiformis* (B) isolated from environmental sources in Kerman city, Southeastern Iran, based on partial 18S rDNA sequencing using maximum likelihood approach. Phylogenetic tree was generated by MEGA 7.0 with bootstrap value of 1000 replicates. Blue circles: water sample. Brown circles: soil sample. (For interpretation of the references to colour in this figure legend, the reader is referred to the web version of this article.)

Phylogenetic analysis revealed that the T4 genotypes of *Acanthamoeba* (*A. polyphaga* and *A. Castellani*) obtained in the present study were co-clustered with T4 genotype of *Acanthamoeba* isolated from Iran, Malaysia, and the USA. Additionally, two isolates of *Acanthamoeba* T3 genotype (*A. griffini*) formed a sister clade with *Acanthamoeba* from Italy, Iran, and the USA. Also in this study, the isolates identified as T5 (*A. lenticulata*) and T6 (*A. palestinensis*) genotypes were in the same cluster as the isolates from Iran, and the USA (Fig. 3).

## 4 Discussion

FLA are a group of protozoa distributed in various environments, and they present a potential threat to human health. Therefore, more research is required to identify the potential sources of FLA contamination. This study represents an investigation of the occurrence and molecular identification of potentially human pathogenic FLA in various environmental sources, including soil, tap water, and fountain water samples within the city of Kerman.

Numerous studies have reported FLA in environmental samples, however most of the studies have been typically focused on a single genus/species of FLA or a specific environment such as water (Armand et al., 2016; Attariani et al., 2020; Eftekhari-Kenzerki et al., 2021; Majid et al., 2017) and soil (Reynaud et al., 2020; Sousa-ramos et al., 2021; Tanzifi et al., 2021). Iran is a large country with quite different ecological and climatic diversity. A small proportion of the studies in Iran have been conducted in the southeastern regions of the country. Table 3 summarizes the current data on the FLA detected in various environmental sources of three provinces in the southeast of the country. In Sistan and Baluchestan province, a study on water samples from pools and ponds, identified three genotypes of *Acanthamoeba* including T3, T4, and T5 (Aghajani et al., 2016). In Kerman province 80 water samples were collected from public swimming pools and reported the presence of T3 and T4 genotypes of *Acanthamoeba* as well as *N.australiensis* (Eftekhari-Kenzerki et al., 2021; Solhjoo et al., 2022). Additionally, a study on the environmental samples in a referral hospital in Kerman city reported the presence of *Acanthamoeba* and *Protacanthamoeba* species (Akhlaghi et al., 2025).

**Table 3:** Summary of the findings of studies on the environmental isolates of free-living amoebae in southeastern Iran.

Province (City)	Sample type	Sample Source	Method		No. samples	Species / Genotypes	References
			Morphology (Culture)	Molecular			
South Khorasan (Qaen)	Water	Squares, Public Park pools, Park fountains	Culture:13/40 *Acanthamoeba* (32.5 %)	18S rDNA PCR: 10/13 *Acanthamoeba* sp. (76.93 %)	40	*–*	(Behravan et al., 2016b)
South Khorasan (Birjand)	Water	Squares, Pools, Park fountains, Water stations	Culture:19/50 *Acanthamoeba* (38 %) 2/50 *Vahlkampfiidae* (4 %)	18S rDNA PCR: 15/19 *Acanthamoeba* sp. (78.9 %)	50	*–*	(Behravan et al., 2016a), (Mahmoodreza et al., 2015)
Sistan and Baluchestan (Zabol, Zahak, Hirmand, Hamoon, and Nimrooz)	Water	Pools, Stagnant water in Ponds	Culture:82/93 FLA (88.17 %)	18S rDNA PCR-sequencing: 38/82 *Acanthamoeba* (46.34 %)	93	34/38 *Acanthamoeba* T4 genotype: (89.47 %) 3/38 *Acanthamoeba* T5 genotype: (7.9 %) 1/38 *Acanthamoeba* T3 genotype: (2.63 %)	(Aghajani et al., 2016)
Kerman (Kerman, Jiroft, Kahnuj)	Water	Public indoor swimming pools	Culture:32/80 *Acanthamoeba* sp. (40 %)	18S rDNA PCR-sequencing: 32/32 *Acanthamoeba* (100 %)	80	2/3 *Acanthamoeba* T3 genotype, 1/3 *Acanthamoeba* T4 genotype	(Eftekhari-Kenzerki et al., 2021)
Kerman (Kerman, Jiroft, Kahnauj)	Water	Public indoor swimming pools	Culture:7/80 *Naegleria* sp.	ITS PCR-sequencing: 7/7 *Naegleria australiensis* (100 %):	80	*Naegleria australiensis*	(Solhjoo et al., 2022)
Kerman (Kerman)	Tap water Soil, Dust, Contact lenses, Lens care solutions	Ophthalmology ward, operating rooms of hospital	Culture:8/70 (11.4 %) FLA	18S rDNA PCR-sequencing: *4/8 Acanthamoeba sp* (50 %) *4/8 Protacanthamoeba bohemica* (50 %)	70	2/4 *Acanthamoeba* T4 genotype: (50 %) 2/4 *Acanthamoeba* sp.: (50 %) *4/8 Protacanthamoeba bohemica:* (50 %)	(Akhlaghi et al., 2025)
Kerman (Kerman)	Water, Soil	Parkes, Squares, Gardens, Fountains, Hospitals, University campus, public swimming pools	Culture:73/141 (51.7 %) FLA	18S and 5.8S rDNA PCR-sequencing:34/43 (79.1 %) *Acanthamoeba: 16/34 (47.1 %), Hartmannella: 16/34 (47.1 %), Vahlkampfia: 2//34 (5.88 %)*	141	8/16 *Acanthamoeba* T4 genotype: *(*50 %) 2/16 *Acanthamoeba T3* genotype: (12.5 %) 2/16 *Acanthamoeba T*6 genotype: (12.5 %) 4/16 *Acanthamoeba* T5 genotype: (25 %) 16/16 *Hartmannella* (*V. vermiformis*): (100 %) 2/2 *Vahlkampfidae:* *1/2 Naegleria australiensis:* (50 %) 1/2 *Naegleria canariensis:* (50 %)	This study

However, there is no data of the occurrence of potentially pathogenic FLA in soil and water samples in Kerman province. Our findings revealed that more than 77 % of fountain water samples tested positive for FLA. In the current study, *Acanthamoeba* spp. were the most detected amoebae (60.7 % and 66.6 %) in fountain and tap water samples. These findings are consistent with previous studies that identified *Acanthamoeba* as the predominant amoeba in surface and tap water in various regions of the world (Sharifi et al., 2022; Yousuf et al., 2013).

Based on the findings of the present study, the most common *Acanthamoeba* isolates were found in fountain water samples belonging to genotype T4 (72.7 %), T3 (18.2 %), and T6 (9.1 %). These findings are consistent with previous studies which reported *Acanthamoeba* T4 genotype as the dominant genotype in water sources of Iran and several other countries (Abd El Wahab et al., 2018; Carnt et al., 2020; Golestani et al., 2018; Nazar et al., 2011; Sharifi et al., 2022). T4 genotype was also found in other environmental sources including soil and tap water samples, as reported in several studies (Abd El Wahab et al., 2018; Badirzadeh et al., 2011; Diehl et al., 2021; Lasjerdi et al., 2011). Varied species of *Acanthamoeba* can cause granulomatous amoebic encephalitis (GAE) and *Acanthamoeba* keratitis (AK). Studies worldwide have indicated that T4 genotype is the most common cause of AK, while, with lower frequency other genotypes (T2, T3, T5, T6, T8, T9, T11, T13, and T15), have also been found in patients with AK (Diehl et al., 2021).

Based on our results T6 genotype was found in tap water samples. This study represents the first documented identification of the T6 genotype in tap water samples from southeastern of Iran. This genotype is reported in Caspian sea coastline seawater samples (Mahmoudi et al., 2021). In Brazil *Acanthamoeba* T2, T4, and T6 genotypes were identified in tap water samples collected from various schools (Winck et al., 2011). In Iran, T3 genotype have been identified in a 35-year-old woman wearing cosmetic soft contact lenses (Niyyati et al., 2010). Also, this genotype was reported from swimming pool and surface waters from southeastern provinces of the country (Aghajani et al., 2016; Eftekhari-Kenzerki et al., 2021). T5 genotype was not found in the water isolates of our study as well as other studies in Kerman province, however studies conducted in other regions in Iran isolated this genotype from surface and tap water samples (Armand et al., 2016; Javanmard et al., 2017). Our study detected *H.* (*Vermamoeba*) *vermiformis* in fountain and tap water samples. Previous studies suggested this amoeba as a reservoir and/or carrier for several pathogenic fungi, viruses, and bacteria in humans including *Legionella pneumophila*. Moreover, the presence of this amoeba has been linked to an increase in the growth of the drug-resistant pathogen, *Stenotrophomonas maltophilia* (Delafont et al., 2018; Siddiqui et al., 2021)*.* In addition to the water samples, different species of *Hartmannella* (*Vermamoeba*) and *Acanthamoeba* were identified in the soil samples collected in the present study. *Vermamoeba vermiformis* was found to be the most abundant FLA in the soil samples in Kerman, that is consistent with the findings of a study by Reynaud reporting this species as the most abundant FLA in Guadeloupe. (Reynaud et al., 2020). The detection of potentially pathogenic FLA, particularly *Acanthamoeba* genotype T4 (with a prevalence of 72.7 %), in both fountain and tap water sources in Kerman underscores a significant public health concern regarding human exposure to severe infections such as *Acanthamoeba* keratitis (AK). The detection of FLA in tap water is especially critical due to its routine use for personal hygiene activities, including washing and bathing, highlighting challenges in current water filtration systems. Moreover, the presence of *Naegleria* species in fountain water raises additional risks associated with aerosol inhalation (Saburi et al., 2017).

Soil contamination with FLA, including pathogenic T5 genotypes, further reflects a global pattern of environmental dissemination, with a reported prevalence of 55.1 % in solid matrices. This contamination increases the possibility of exposure through airborne dust particles (Chaúque et al., 2023). Consequently, there is a potential risk of transmission of FLA through soil exposure, particularly among children and individuals with frequent occupational contact such as gardeners and agricultural workers who frequently handle soil.

There are several studies on the detection of FLA from soil samples (Golestani et al., 2018; Karamati et al., 2016; Reynaud et al., 2020; Sousa-ramos et al., 2021; Tanzifi et al., 2021; Xuan et al., 2017). In our study *Acanthamoeba* species were the second most isolated species from soil, all of which belonged to the T5 genotype. Tanzifi et al. found that the frequency of *Acanthamoeba, V. vermiformis*, and *Naegleria* in the soil samples from public places in Northern Iran, is 52.4 %, 45.9 %, and 1.6 %, respectively. They also demonstrated that all isolated *Acanthamoeba* belonged to the T4 genotype (Tanzifi et al., 2021). Soil samples collected from Santiago Island, Cape Verde showed a prevalence of 82.4 % for *Acanthamoeba* spp., and 5.9 % for each of *V. vermiformis*, *Stenamoeba dejonckheerei*, and *Vannella pentlandii*. The T4 genotype was the most common (13/14; 92.9 %), followed by the T5 genotype (1/14; 7.1 %) (Sousa-ramos et al., 2021). Another study identified *Acanthamoeba* spp. in 41.6 % of the soil samples. Genotyping of the soil isolates revealed the presence of T3, T4, T5, and T11 genotypes in the northwest of Iran (Karamati et al., 2016). Previous studies have identified the T5 genotype in various environmental samples, including seawater, biofilms, fresh vegetables, soil, and tap water (Fatemi et al., 2022; Lasjerdi et al., 2011; Mahmoudi et al., 2021; Xuan et al., 2017). Additionally, the T5 genotype has been isolated from keratitis patients, indicating the potential risk of this genotype to humans (Spanakos et al., 2006).

Two *Naegleria* species, *N. canariensis* and *N. australiensis*, were detected in fountain water samples in Kerman. Previous studies identified several species of *Naegleria* in various environmental sources (Edagawa et al., 2009; Javanmard et al., 2017; Reynaud et al., 2020; Sharifi et al., 2022). Until now, only *N. fowleri* has been recognized as a human pathogen among at least 47 species within the genus *Naegleria* (Latifi et al., 2017). Our findings showed that *Acanthamoeba* and *Hartmannella* are more frequent compared to *Naegleria* species. The high frequency of *Acanthamoeba* and *Hartmannella* species in environmental samples may be attributed to the resistance of their cysts to unfavorable environmental conditions (Al-Herrawy et al., 2017). Chlorination is widely used as a method for disinfecting water supplies (Tsitsifli and Kanakoudis, 2018). Personal preventive measures and monitoring of water quality should be considered as this method cannot be adequately effective against potentially pathogenic FLA in water supplies and swimming pools.

A meta-analysis of 38 studies conducted in Iran reported an overall isolation rate of 36 % for FLA from water samples, with *Acanthamoeba* particularly the pathogenic T4 genotype emerging as the most frequently isolated species. This national trend closely parallels our results in Kerman, where genotype T4 was predominant in both fountain and tap water sources (Saburi et al., 2017). At the global level, a systematic review encompassing 103 studies from 35 countries estimated the pooled prevalence of *Naegleria* spp. in aquatic environments at 26.4 %, with *N. fowleri*, the causative agent of primary amoebic meningoencephalitis. Although *N. fowleri* was not identified in our samples, the presence of other *Naegleria* species underscores regional ecological variation and highlights the importance of continued environmental monitoring (Saberi et al., 2020). In wastewater settings, the global prevalence of FLA has been reported at 69 %, with *Acanthamoeba* (47.5 %) and *Vermamoeba* (28.2 %) representing the most common genera (da Silva et al., 2024). These findings are consistent with our soil data, where *Vermamoeba* was the dominant isolate. Similarly, a meta-analysis of FLA in recreational water sources, including swimming pools, revealed a global prevalence of 44.3 % (Chaúque et al., 2022), which aligns with the high detection rates observed in our fountain and tap water samples. Finally, a global review of FLA in solid matrices such as soil, dust, and sediment reported an overall prevalence of 55.1 %, with *Acanthamoeba* (52.2 %) and *Vermamoeba* (36.1 %) again being the most frequently identified genera (Chaúque et al., 2023) The predominance of *Vermamoeba* and *Acanthamoeba* genotype T5 in our soil samples corresponds with these global patterns, although the absence of genotype T4 in Kerman soil may reflect localized environmental or climatic influences.

Public education campaigns are essential to raise awareness about the risks posed by FLA in water and soil environments. Schools should incorporate targeted lessons to inform students about safe hygiene practices and the risks associated with swimming in open water bodies. High-risk groups, such as contact lens users, require specific guidance on proper lens cleaning, the use of standard disinfectant solutions, and avoidance of non-sterile water exposure. Also, immunocompromised individuals should be educated on minimizing environmental exposure and adhering to strict personal hygiene protocols. Training healthcare providers and caregivers further strengthens preventive strategies by ensuring consistent risk communication. In addition, educating and raising awareness among medical staff enhances their ability to recognize early symptoms, deliver timely interventions, and effectively communicate potential risks to patients. Collectively, these educational interventions reduce infection risk and promote community-wide awareness against amoebic contamination. To mitigate the risks and reduce the possibility of infections caused by FLA, public health initiatives must emphasize behavioral modifications, such as discouraging diving in warm recreational waters known to harbor FLA (Chaúque et al., 2022; Saberi et al., 2020).

Both globally and within Iran, ensuring water safety necessitates the adoption of advanced disinfection strategies beyond conventional chlorination, which remains ineffective against the resistant cyst forms of FLA (Chaúque et al., 2022). Finally, further research and comprehensive surveillance programs should be expanded to encompass previously unexamined provinces and incorporate sediment sampling from potable water storage tanks to enhance detection and control efforts (Chaúque et al., 2023; Saburi et al., 2017).

## 5 Conclusion

The current study showed the distribution of FLA in different environmental sources in Southeastern of Iran. Four *Acanthamoeba* genotypes as well as two *Naegleria* species, *Hartmannella* (*Vermamoeba) vermiformis* and *Balamuthia mandrillaris*, were found in water and soil samples. The results revealed high frequency of potentially pathogenic FLA in various environmental sources in southeastern Iran. The extensive presence of FLA in environmental samples increases the risk of human and animal infection. Therefore, for a safe environment, appropriate measures should be taken to manage the potential risk of FLA in various environmental sources. Given the high prevalence of potentially pathogenic FLA, particularly *Acanthamoeba* T4, and their resistance to conventional chlorination, effective risk mitigation requires advanced water disinfection methods, routine environmental monitoring, public education and the installation of warning signs in recreational areas to reduce human exposure.

The following are the supplementary data related to this article.

Supplementary Table 1 PCR thermal cycling profile used for DNA amplification of different species of free-living amoebae isolated from environmental sources in Kerman, Southeastern Iran. Supplementary Table 1

## CRediT authorship contribution statement

**Abbas Ali Ghasemi:** Writing – review & editing, Writing – original draft, Visualization, Validation, Software, Methodology, Investigation, Formal analysis, Data curation, Conceptualization. **Elham Akhlaghi:** Writing – review & editing, Writing – original draft, Visualization, Validation, Software, Investigation, Formal analysis, Data curation. **Zahra Babaei:** Writing – review & editing, Validation, Methodology, Investigation, Formal analysis. **Naser Zia-ali:** Writing – review & editing, Validation, Methodology, Investigation. **Sima Rostami:** Writing – review & editing, Software, Formal analysis, Data curation. **Mehdi Borhani:** Writing – review & editing, Writing – original draft, Investigation, Data curation. **Tayyebeh Ghasemi:** Writing – review & editing, Writing – original draft, Data curation. **Majid Fasihi Harandi:** Writing – review & editing, Writing – original draft, Visualization, Validation, Supervision, Resources, Project administration, Methodology, Investigation, Funding acquisition, Formal analysis, Conceptualization.

## Consent to participate

Not applicable.

## Consent for publication

Not applicable.

## Ethical approval

Not applicable.

## Funding

This research was financially supported by the Vice-Chancellor for Research and Technology, 10.13039/501100004621Kerman University of Medical Sciences, Grant No. 90.76.

## Declaration of competing interest

The authors declare that they have no known competing financial interests or personal relationships that could have appeared to influence the work reported in this paper.

## Data Availability

The sequences of 18S rRNA and 5.8S rRNA were deposited in the NCBI GenBank with the accession numbers KC694162- KC694192 and OP218467- OP218468.
