# Supplementary material for: Molecular identification of potentially pathogenic free-living amoebae in environmental samples from urban areas of Kerman, Southeastern Iran
Source: Parasite Epidemiol Control. 2026 Jan 3;32:e00476. doi: 10.1016/j.parepi.2026.e00476 (PMC12811234; doi:10.1016/j.parepi.2026.e00476)
Supplement: Supplementary Table 1 — PCR thermal cycling profile used for DNA amplification of different species of free-living amoebae isolated from environmental sources in Kerman, Southeastern Iran. [file mmc2.docx]

**Supplementary Table 1.** PCR thermal cycling profile used for DNA amplification of different species of free-living amoebae isolated from environmental sources in Kerman, Southeastern Iran.

| **Species** | **Step** | **Time** | **Temperature (°C)** | **Cycles** |
| --- | --- | --- | --- | --- |
| ***Acanthamoeba* spp** | First Denaturation | 7 min | 95 | 1 |
|  | Denaturation | 1 min | 95 | 40 |
|  | Annealing | 1 min | 58 |  |
|  | Extension | 1 min | 72 |  |
|  | Final Extension | 15 min | 72 | 1 |
| ***Hartmannella* spp** | First Denaturation | 1 min | 94 | 1 |
|  | Denaturation | 35 s | 94 | 35 |
|  | Annealing | 45 s | 50 |  |
|  | Extension | 1 min | 72 |  |
|  | Final Extension | 10 min | 72 | 1 |
| ***Vahlkampfiidae*** | First Denaturation | 2 min | 95 | 1 |
|  | Denaturation | 30 s | 95 | 30 |
|  | Annealing | 30 s | 50 |  |
|  | Extension | 30 s | 72 |  |
|  | Final Extension | 5 min | 72 | 1 |
| ***Balamuthia* spp** | First Denaturation | 7 min | 95 | 1 |
|  | Denaturation | 1 min | 95 | 40 |
|  | Annealing | 1 min | 58 |  |
|  | Extension | 1 min | 72 |  |
|  | Final Extension | 15 min | 72 | 1 |
